# CMR evaluation of change in myocardial strain following transcatheter aortic valve implantation

**DOI:** 10.1186/1532-429X-16-S1-P260

**Published:** 2014-01-16

**Authors:** Akhlaque Uddin, Timothy A Fairbairn, Peter P Swoboda, Ananth Kidambi, Manish Motwani, David P Ripley, Tarique A Musa, Adam K McDiarmid, Sven Plein, John P Greenwood

**Affiliations:** 1 Academic Unit of Cardiovascular Medicine, Multidisciplinary Cardiovascular Research Centre (MCRC) & The Division of Cardiovascular and Diabetes Research, Leeds Institute of Genetics, Health and Therapeutics, Leeds, UK

## Background

Transcatheter Aortic Valve Implantation (TAVI) is the treatment of choice for patients with severe symptomatic aortic stenosis (AS) who are at high surgical risk. AS results in changes in myocardial strain and twist. Myocardial strain, strain rate and twist can be measured with myocardial tagging CMR. It is not known how TAVI affects LV diastolic and systolic function as measured by CMR tagging.

## Objective

To determine changes in myocardial strain following TAVI.

## Methods

25 patients (age 80 ± 6 years, male 14 (56%), EuroSCORE 22 ± 14) underwent CMR (1.5T, Intera CV, Philips Healthcare) before and 6 months after TAVI. Tagged cine images were acquired at the apex, mid-ventricle and base with a complementary spatial modulation of magnetization (CSPAMM) pulse sequence (FOV 300 mm, matrix 128 × 128, slice thickness 10 mm, tag separation 8 mm, 18 phases, typical TR/TE 30 ms/6 ms, flip angle 25°). Data were analysed using inTag^© ^software (Creatis, Lyon, Fr). Endocardial and epicardial contours were drawn and segmented into 3 layers.

## Results

Following TAVI, peak Lagrangian circumferential strain increased in the mid-LV (-0.19 ± 0.06 vs. -0.22 ± 0.07, P = 0.03). There was no significant change in apical (-0.20 ± 0.06 vs. -0.20 ± 0.07, P = 0.81) or basal (-0.19 ± 0.06 vs. -0.20 ± 0.06, P = 0.24) circumferential strain. LV twist decreased after TAVI (17.2 ± 4.9° vs. 13.9 ± 5.4°, P = 0.04) and peak systolic strain rate increased (-0.92 ± 0.24S^-1 ^vs. -1.11 ± 0.23S^-1^, P = 0.001) but there was no change in early diastolic strain rate (0.91 ± 0.51S^-1 ^vs. 0.88 ± 0.43S^-1^, P = 0.98).

## Conclusions

TAVI results in an improvement in mid-LV circumferential strain, and a decrease in myocardial twist. Systolic strain rate increased following TAVI but there was no significant change in diastolic strain rate. This suggests that whilst systolic function improves, diastolic function does not improve in severe AS at 6 months post-TAVI.

## Funding

SP is funded by a British Heart Foundation fellowship (FS/10/62/28409). SP and JPG receive an educational research grant from Philips Healthcare.

**Figure 1:**
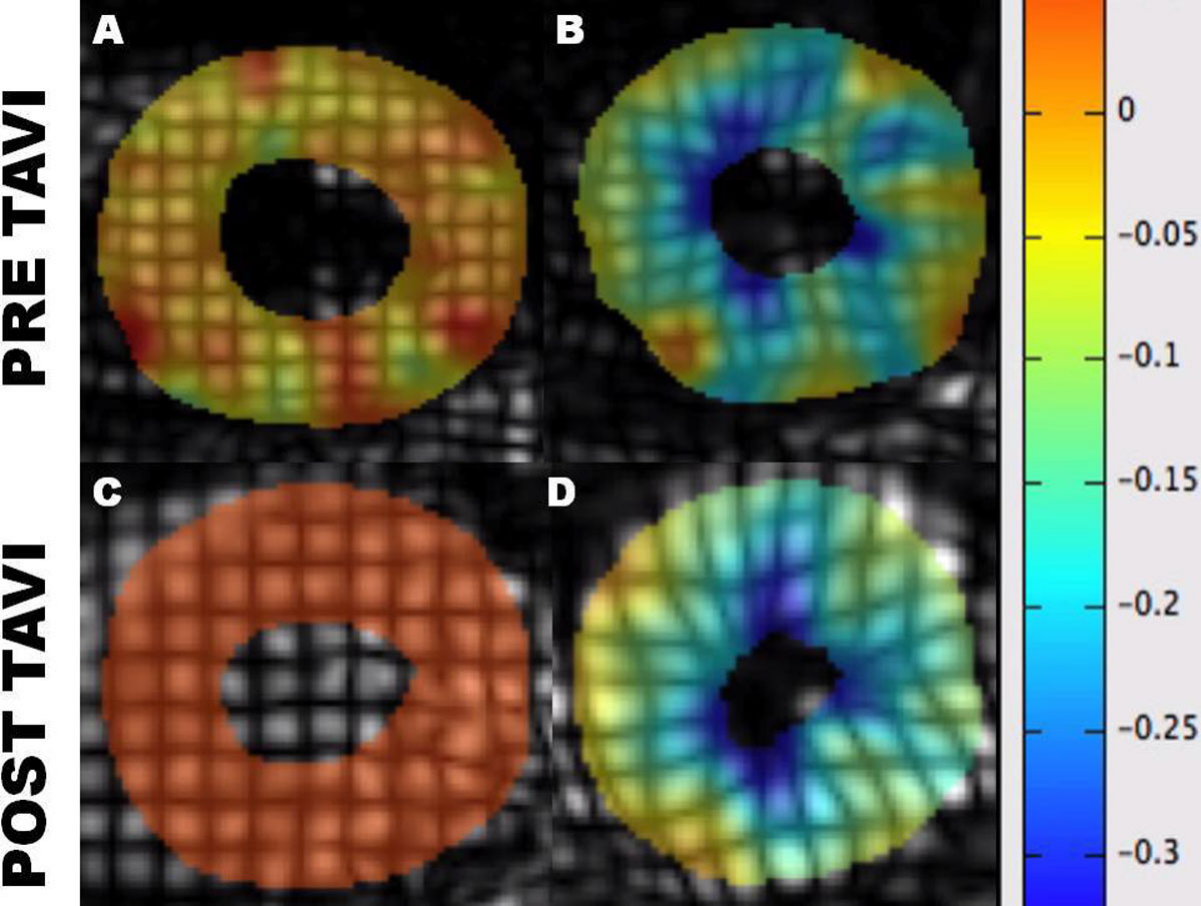
**Example of inTag analysis using complementary spatial modulation of magnetization (CSPAMM)**. Diastole (A, C) and systole (B, D).

**Figure 2:**
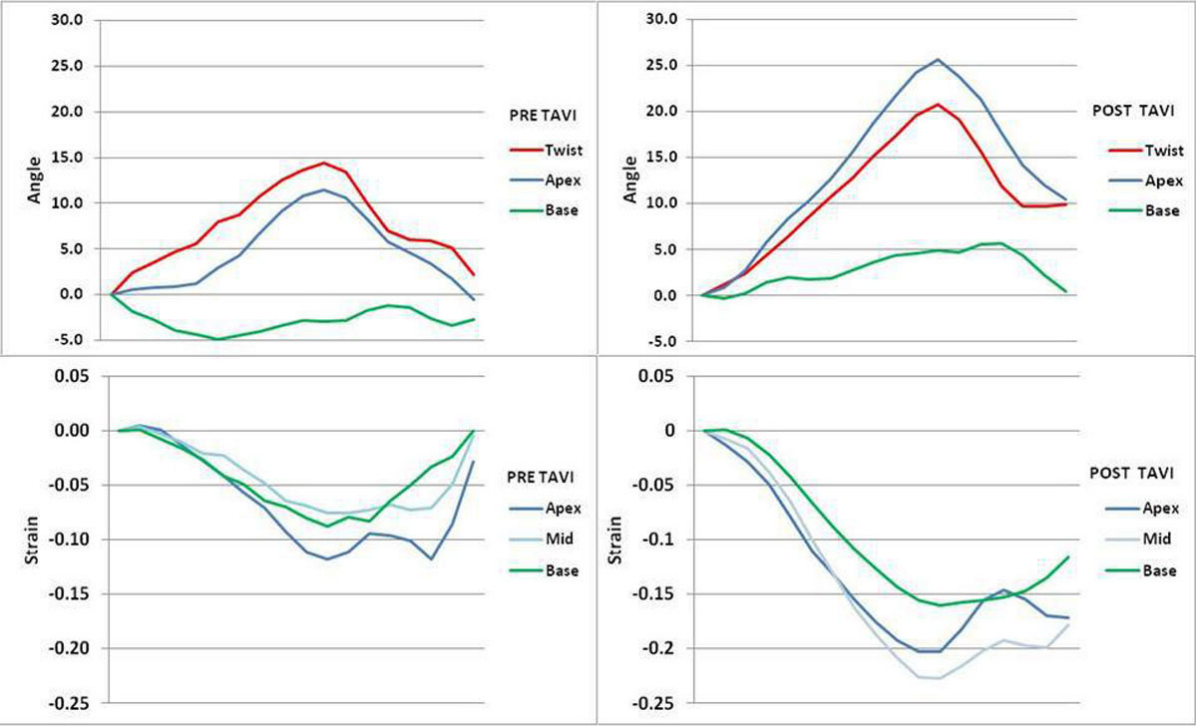
**Change in twist and circumferential strain**. A) Twist pre-TAVI. B) Twist post TAVI. Circumferential strain pre- (C) and post-TAVI (D).

